# *In vitro *susceptibility to pyrimethamine of DHFR I164L single mutant *Plasmodium falciparum*

**DOI:** 10.1186/1475-2875-10-283

**Published:** 2011-09-27

**Authors:** Valérie Andriantsoanirina, Rémy Durand, Bruno Pradines, Eric Baret, Christiane Bouchier, Arsène Ratsimbasoa, Didier Ménard

**Affiliations:** 1 Unité de Recherche sur le Paludisme, Institut Pasteur, Antananarivo, Madagascar; 2 Laboratoire de Parasitologie-Mycologie, Hôpital Avicenne, 125 Rue de Stalingrad 93000 Cedex 8, Bobigny France; 3 Unité de Parasitologie UMR6236, Institut de Recherche Biomédicale des Armées, Marseille, France; 4 Plateforme Génomique, Institut Pasteur, Paris, France; 5 Ministère de la Santé, du Planning Familial et de la Protection Sociale, Antananarivo, Madagascar; 6 Unité d'Epidémiologie Moléculaire, Institut Pasteur, Phnom Penh, Cambodge

## Abstract

**Background:**

Recently, *Plasmodium falciparum *parasites bearing *Pfdhfr *I164L single mutation were found in Madagascar. These new mutants may challenge the use of antifolates for the intermittent preventive treatment of malaria during pregnancy (IPTp). Assays with transgenic bacteria suggested that I164L parasites have a wild-type phenotype for pyrimethamine but it had to be confirmed by testing the parasites themselves.

**Methods:**

Thirty *Plasmodium falciparum *clinical isolates were collected in 2008 in the south-east of Madagascar. A part of *Pfdhfr *gene encompassing codons 6 to 206 was amplified by PCR and the determination of the presence of single nucleotide polymorphisms was performed by DNA sequencing. The multiplicity of infection was estimated by using an allelic family-specific nested PCR. Isolates that appeared monoclonal were submitted to culture adaptation. Determination of IC_50s _to pyrimethamine was performed on adapted isolates.

**Results:**

Four different *Pfdhfr *alleles were found: the 164L single mutant-type (N = 13), the wild-type (N = 7), the triple mutant-type 51I/59R/108N (N = 9) and the double mutant-type 108N/164L (N = 1). Eleven out 30 (36.7%) of *P. falciparum *isolates were considered as monoclonal infection. Among them, five isolates were successfully adapted in culture and tested for pyrimethamine *in vitro *susceptibility. The wild-type allele was the most susceptible with a 50% inhibitory concentration (IC_50_) < 10 nM. The geometric mean of IC_50 _of the three I164L mutant isolates was 6-fold higher than the wild-type with 61.3 nM (SD = 3.2 nM, CI95%: 53.9-69.7 nM). These values remained largely below the IC_50 _of the triple mutant parasite (13,804 nM).

**Conclusion:**

The IC_50_s of the I164L mutant isolates were significantly higher than those of the wild-type (6-fold higher) and close from those usually reported for simple mutants S108N (roughly10-fold higher than wild type). Given the observed values, the determination of IC_50_s directly on parasites did not confirm what has been found on transgenic bacteria. The prevalence increase of the *Pfdhfr *I164L single mutant parasite since 2006 could be explained by the selective advantage of this allele under sulphadoxine-pyrimethamine pressure. The emergence of highly resistant alleles should be considered in the future, in particular because an unexpected double mutant-type allele S108N/I164L has been already detected.

## Background

*Plasmodium falciparum *malaria remains a major cause of morbidity and mortality in endemic areas, affecting mainly African children under five years of age and pregnant women [1]. Currently, in these areas, artemisinin combinations therapy (ACT) is recommended as first-line treatment for uncomplicated *P. falciparum *malaria, while the intermittent preventive treatment of malaria in pregnancy (IPTp) relies on the administration of antifolate sulphadoxine-pyrimethamine (SP) combination. Anti-malarial drugs used for IPTp must be efficacious, safe, tolerable, cheap, and easy to administer, preferably as a single dose [2]. So far, SP is the only drug which has these attributes, despite its decreasing efficiency [3].

Analysis of the molecular basis of anti-malarial drug resistance has demonstrated that mutations in the dihydrofolate reductase (*dhfr*) and dihydropteroate synthase genes are associated with development of SP resistance. Resistance to pyrimethamine (PYR) is due to point mutations in the *P. falciparum *dihydrofolate reductase gene (*Pfdhfr*) [4-9] and the *Pf*DHFR S108N point mutation is the main molecular event, followed by stepwise selection of additional mutations in other positions (codons 51, 59 and 164). Several studies have already demonstrated that parasites with a triple-mutant allele (N51I/C59R/S108N) have markedly reduced *in vitro *susceptibility to PYR, and the presence of the triple-mutation allele increases the risk of SP therapeutic failure [10]. An additional mutation, I164L, confers to the quadruple mutant a high level of resistance to PYR [11], abrogating the clinical efficacy of SP as observed in Southeast Asia and South America [12] but rarely in Africa [13].

In African countries and the Comoros Island, three haplotypes including the I164L allele have been described: both triple mutants N51I/S108N/I164L and C59R/S108N/I164L, and the quadruple mutant [14,15]. In 2006, a novel I164L single mutant in one field isolate in Madagascar was observed [16]. In 2008, the *Pfdhfr *I164L polymorphism was prevalent in southern sentinel sites of the Great Island with 8.8% of isolates carrying this mutation [14]. Microsatellite markers analysis showed that this unique allele emerged independently in these sites. Until now, the *Pfdhfr *I164L allele has not been reported elsewhere in the world. Previous published data have also shown that the triple mutant N51I/C59R/S108N have largely spread in Madagascar but the quadruple mutant has not been reported so far.

In 2009, Lozovsky *et al *have explored and analysed the possible evolutionary pathways of PYR resistance using experimental systems with transgenic bacteria in which all possible mutational intermediates were created by site-directed mutagenesis (N51I, C59R, S108N, and I164L) in *Pf*DHFR enzyme [17]. Their findings suggested that *P. falciparum *parasites harbouring single mutant *Pfdhfr *I164L (IC_50 _= 0.29 μg/ml) were as sensitive to PYR as the wild type allele (0.27 μg/ml). This data was not consistent with the observation of the rapid rise in the prevalence of parasites with the single 164L mutation in Madagascar following the massive use of the SP [14].

In this context, the main objective of the study was to assess the PYR *in vitro *susceptibility of parasites themselves and to compare those harboring the single 164L mutation with those harbouring wild-type and triple-mutant *Pfdhfr *alleles in order to decipher the epidemiological characteristics of this haplotype.

## Methods

### Sample collection

*Plasmodium falciparum *clinical isolates were collected in 2008 from patients seeking treatment for malaria at Farafangana Health Centre in the south-east of Madagascar where high prevalence of *Pfdhfr *I164L single mutant allele was previously observed [18]. All patients with fever were screened with the CareStart^© ^rapid diagnostic test (AccessBio^©^). Giemsa-stained thin and thick blood films were prepared for each patient with a positive rapid diagnostic test result. The various species of *Plasmodium *were identified and parasitaemia was assessed by a skilled microscopist. Once informed consent had been obtained from all adults and from at least one parent for minors, 5 ml of venous blood were collected on EDTA tube. Patients with positive microscopy results were promptly treated according to National Malaria Policy. Blood samples were sent to Institut Pasteur, Antananarivo, at +4°C within 24 to 48 h of collection. Giemsa-stained thin blood smears were examined to check for mono-infection with *P. falciparum *and to determine parasite density. *Plasmodium falciparum *samples were split into two different aliquots: cryopreserved aliquots stored in liquid nitrogen for culture adaptation and *in vitro *assays, and fresh blood aliquots stored at -20°C until genomic DNA extraction.

### Genomic DNA extraction

DNA was extracted from initial infected blood aliquots and from parasites obtained after culture adaptation, by the phenol-chloroform method [19].

### *Plasmodium *species molecular diagnosis

Parasite species were confirmed in both DNA extracts by real-time PCR, using species-specific primers as described by de Monbrison [20] with a protocol adapted for the RotorGene^® ^3000 thermocycler (Corbett Life Science^®^, Sydney, Australia).

### *Pfdhfr *amplification & sequencing

A 600 bp fragment of *Pfdhfr *gene encompassing codons 6 to 206 was amplified by nested PCR technique on both DNA extracts, as previously described [21]. Sequencing reactions were carried out with a ABI Prism BigDye Terminator cycle sequencing ready reaction kit and were run on a model 3730 xl genetic analyzer (Applied Biosystems, Courtaboeuf, France). Electrophoregrams were visualized and analyzed with CEQ2000 genetic analysis system software (Beckman Coulter, Villepinte, France). The amino acid sequences were compared with the 3D7 *Pf*DHFR wild-type amino acid sequence (PFD0830w, GenBank accession number AL844503). The presence of single nucleotide polymorphisms was confirmed by reading both the forward and the reverse strands. Parasites with mixed alleles (in which both wild-type and mutant alleles were present) were considered mutants. Haplotypes for drug resistance markers were reconstructed from the full sequence presenting an unambiguous single allele signal at all positions.

### Multiplicity of infection (MOI)

The multiplicity of infection, defined as the highest number of alleles detected at either of the two loci, was estimated by using an allelic family-specific nested PCR (MAD20, K1, and RO33 for *Pfmsp-1 *and 3D7 and FC27 for *Pfmsp-2*), as described previously (1). All PCR amplifications of initial infected blood aliquots contained a positive control (genomic DNA from strains W2, HB3, and 3D7) and a negative control (no target DNA). Only samples showing monoclonal *P. falciparum *infections were selected for culture adaptation.

### Culture adaptation and *in vitro *susceptibility assays

Aliquots preserved in liquid nitrogen were sent to IRBA, Marseille, France, for culture adaptation and assessment of 50% inhibitory concentration (IC_50_) to PYR. PYR was obtained from Sigma (St. Louis, MO). Stock solution was prepared in ethanol. Twofold serial dilutions were prepared in sterile water and distributed in triplicate into Falcon 96-well flat-bottomed plates (Becton Dickinson, Franklin Lakes, NJ). The 3D7 clone (wild-type *Pfdhfr *allele) and the W2 Indochina clone (triple mutant-type *Pfdhfr *allele) were obtained from MR4-ATCC (Manassas, VA, USA) and used as controls to test the batch of plates.

Determination of the *in vitro *susceptibility was performed in triplicate on culture adapted isolates and on references strains, using the *in vitro *isotopic microtest as previously described [22]. The 50% inhibitory concentration (IC_50_), i.e., the drug concentration corresponding to 50% of the uptake of [^3^H] hypoxanthine by the parasites in drug-free control wells, was determined by non-linear regression analysis of log dose-response curves (Riasmart; Packard, Meriden, NJ). Data were analysed after logarithmic transformation and expressed as the geometric mean IC_50_, and 95% confidence intervals were calculated (Stata 9; StataCorp LP, Texas, USA).

### Ethical approval

The study protocol was reviewed and approved by the Ethics Committee of the Ministry of Health of Madagascar (N°007/SANPF/2007). An informed written consent was provided by the parents/guardians of all patients before they were included in the study.

## Results

Thirty *P. falciparum *samples were collected in a four-day mission with parasitaemia ranging from 0.01 to 0.72%. Among them, sequencing of the *Pfdhfr *gene revealed four different alleles: the 164L single mutant-type (N = 13), the wild-type (N = 7), the triple mutant-type 51I/59R/108N (N = 9) and the double mutant-type 108N/164L (N = 1). The latter allele was observed for the first time in this area.

Genotyping of collected isolates showed that 11/30 (36.7%) of *P. falciparum *isolates had a single allelic form and were considered as monoclonal infection: 164L single mutant-type allele (4/13), wild-type allele (2/7), triple mutant-type 51I/59R/108N allele (4/9) and double mutant-type allele 108N/164L (1/1). Among them, five isolates were successfully adapted in culture (1 isolate with wild-type allele, three isolates with 164L single mutant-type allele and one isolate with triple mutant-type 51I/59R/108N allele) and tested for PYR *in vitro *susceptibility. On these samples, *Pfdhfr *genotypes and MOI which were assessed after the culture adaptation period were identical to initial samples.

Pyrimethamine IC_50_s were significantly different among the three *Pfdhfr *alleles (P < 0.001). The wild-type allele was the most susceptible with an IC_50 _< 10 nM. The geometric mean of IC_50 _of the three I164L mutant isolates was significantly higher (6-fold, P < 0.001) than the wild-type with 61.3 nM (SD = 3.2 nM, CI95%: 53.9-69.7 nM). These values remained largely below the IC_50 _of the triple mutant parasite tested in the assay which was 13,804 nM.

## Discussion & Conclusion

The main result of this study was that I164L mutant isolates did not show fully susceptible IC_50_s, but values that were significantly higher than those of the wild-type (six-fold higher). These values were nearer to what is usually reported for simple mutants S108N (roughly10-fold higher than wild type) [23]. Thus, the determination of IC_50_s directly on parasites did not confirm on that specific point what has been found by Lozowski *et al *using transgenic bacteria. The adaptation of isolates to culture conditions is a delicate process, which is not always successful. In the present study, three clonal infections by parasites harbouring the I164L mutations were adapted, which enabled us to observe the low SD of the IC_50_s to PYR between these strains. Given the observed values of IC_50_s, the presence of *Pfdhfr *I164L single mutant parasites did not challenge the current use of SP for IPTp in Madagascar. Indeed, I164L mutant isolates could not been considered as resistant because the usual threshold for PYR resistance was defined as > 2,000 nM, the intermediate values, 100-2000 nM, representing moderate resistance [24]. However, the emergence of highly resistant alleles should be considered as possible for two reasons: first, the prevalence of the I164L allele has increased since year 2006 (14 isolates out 30 (46.7%) in the present series) and second, the presence of the double mutant-type allele S108N/I164L has been detected in a monoclonal isolate. The latter means that the 108N/164L genotype has been formed either on a background of S108N or I164L. This pathway was not expected according to the model of Lozovsky *et al*, but obviously it occurred in the real life. As I164L simple mutant genotype is not found today in another area in the world, Malagasy parasites may have a particular background, which enabled the emergence of this double mutant genotype. A highly resistant IC_50 _(103 μg/ml) for the S108N/I164L genotype was found using transgenic bacteria [17]. Unfortunately, the S108N/I164L isolate was not successfully adapted to culture and it was consequently impossible to confirm this *in vitro *resistance level. Interestingly, these authors reported that the low fitness of the quadruple mutant in the absence of PYR was not observed in transgenic bacteria having the S108N/I164L genotype. If these characteristics were to apply to natural parasites, it could have implications in terms of transmission of these strains. The triple mutant-type 51I/59R/108N was also largely prevalent (9 out 30 (30%) isolates) in the area and may represent a possible background for the emergence of the quadruple mutant. In addition, the diffusion of the quadruple mutant from the Comoros Islands remains possible and could be a source of highly resistant parasites. Combinations of DHFR and DHPS inhibitors act synergistically. To further document the impact of the *Pfdhfr *I164L mutation in SP resistance it may be interesting to determine polymorphisms in *Pfdhps *gene, which was not done for isolates of the present series. A previous work performed in a large series in 2007 in Madagascar showed that 46% of isolates harboured the *Pfdhps *A437G mutation [14]. For all these reasons, epidemiological surveillance is needed in the future to monitor the use of SP in IPTp.

## Competing interests

The authors declare that they have no competing interests.

## Authors' contributions

VA, RD and DM developed the study protocol, oversaw the implementation of the field work and the PCR analysis, and assisted with the data analysis and drafting of the manuscript. AR contributed to the field work. EB performed culture adaptation and in vitro assays. VA undertook real time and nested PCR assays. CB carried out sequencing. BP helped to write the manuscript and gave constructive advice. All authors read and approved the final manuscript.
